# New Appliances

**Published:** 1899-04-01

**Authors:** 


### NEW APPLIANCES.

### KRONTHAL WATERS.

We regret that, owing to a printer's error in the description of these waters, which we gave on March 18th, the colours of the labels were transposed. The water bearing the blue label is intended purely as a table water, whilst that bearing the red label has certain medical properties.

